# Spatiotemporal material functionalization via competitive supramolecular complexation of avidin and biotin analogs

**DOI:** 10.1038/s41467-019-12390-4

**Published:** 2019-09-25

**Authors:** Tom Kamperman, Michelle Koerselman, Cindy Kelder, Jan Hendriks, João F. Crispim, Xandra de Peuter, Pieter J. Dijkstra, Marcel Karperien, Jeroen Leijten

**Affiliations:** Faculty of Science and Technology, Technical Medical Centre, Department of Developmental BioEngineering, University of Twente, Drienerlolaan 5, 7522 NB Enschede, The Netherlands

**Keywords:** Bioinspired materials, Biomaterials - cells, Drug delivery, Tissue engineering

## Abstract

Spatiotemporal control over engineered tissues is highly desirable for various biomedical applications as it emulates the dynamic behavior of natural tissues. Current spatiotemporal biomaterial functionalization approaches are based on cytotoxic, technically challenging, or non-scalable chemistries, which has hampered their widespread usage. Here we report a strategy to spatiotemporally functionalize (bio)materials based on competitive supramolecular complexation of avidin and biotin analogs. Specifically, an injectable hydrogel is orthogonally post-functionalized with desthiobiotinylated moieties using multivalent neutravidin. In situ exchange of desthiobiotin by biotin enables spatiotemporal material functionalization as demonstrated by the formation of long-range, conformal, and contra-directional biochemical gradients within complex-shaped 3D hydrogels. Temporal control over engineered tissue biochemistry is further demonstrated by timed presentation and sequestration of growth factors using desthiobiotinylated antibodies. The method’s universality is confirmed by modifying hydrogels with biotinylated fluorophores, peptides, nanoparticles, enzymes, and antibodies. Overall, this work provides a facile, cytocompatible, and universal strategy to spatiotemporally functionalize materials.

## Abstract (web summary)

Controlled patterning of functionality within hydrogels typically involves complex chemistry. Here, the authors report on a simple competitive binding strategy using avidin and biotin analogs in an injectable biomaterial for spatiotemporally controlled presentation of biochemical stimuli to cells.

## Introduction

Native tissues are spatiotemporally organized structures that undergo constant biochemical and biomechanical modifications. The dynamic nature of our tissues is particularly apparent during major life events, such as fetal development, wound healing, and aging^1–3^. The timed adaptation of the local extracellular matrix is key to these processes, as it directly controls the behavior of cells within tissues^4^. To recapitulate these complex and dynamic behaviors in engineered tissues, accurate spatiotemporal control over a biomaterial’s biochemical composition is required^5^.

Biomaterials that can be controlled in both space and time offer unique opportunities to guide cell behaviors such as adhesion, spreading, migration, survival, and differentiation^6–13^. Currently, the spatiotemporal modification of biomaterials mostly relies on photoresponsive strategies that provide 2.5/3D spatial control over transparent materials. However, these are not compatible with opaque materials, often demand high-end infrastructure, and associate with cytotoxic factors, such as UV-light and radical-based reactions^5,14,15^. For example, from the limited number of approaches available, radical-based involving acrylates and thiol-enes are proven to adversely affect cell viability^16^. Furthermore, 3D spatiotemporal hydrogel patterning by uncaging coumarin-caged thiols inducing a cytocompatible thiol–Michael addition click reaction is technically challenging and difficult to scale due to the use of two-photon technologies^17^. Alternatively, spatiotemporal control via thiol–Michael addition reactions can be achieved using photogenerated bases^18^. However, this strategy is not (cyto)compatible with engineered tissues, where pH values must be maintained within the physiological range. Alternatively, a few in situ biomaterial functionalization methods based on reversible physical interactions have been utilized, which include host–guest interactions and molecular recognition of oligonucleotide or oligopeptide aptamers. Specifically, explored physical interactions to this end rely on complicated and laborious ligand modification strategies, including the modification of ligands with adamantanes^19^, aptamers^20,21^, or leucine zipper domains^22^, which are not part of the standard biochemistry toolbox of most laboratories, which has prevented their widespread use in the biomedical domain. Consequently, an alternative strategy to spatiotemporally modify biomaterials in a cytocompatible, widely available, and translatable manner has remained wanted. We hypothesized that the avidin and biotin-binding pair provides a potential solution to this end.

Biotinylation is one of the most versatile, commonly applied, and readily available modifications in life science^23^. The supramolecular avidin/biotin complex has, among others, been used to endow a material with bioactive moieties in a facile, orthogonal, and cytocompatible manner^12,17,24,25^. Biotin has a non-sulfur containing analog, desthiobiotin, which also specifically interacts with avidin and its analogs, but with a binding affinity that is approximately an order of magnitude lower than biotin (*K*_d,biotin–avidin_ ~ 10^−15^ M versus *K*_d,desthiobiotin–avidin_ ~ 10^−14^ M in solution)^26^. The difference between these binding affinities has, for example, been used to achieve the displacement of desthiobiotin by biotin for the reversible labeling and affinity-based isolation of proteins^27,28^. This primes avidin and biotin analogs as ideal candidate pairs for a tunable biomaterial platform strategy.

Here we pioneer a combination of avidin and biotin analogs to enable mild, specific, and spatiotemporal modification of biomaterials in a straightforward manner. Specifically, we use a combination of neutravidin, biotin, and desthiobiotin to functionalize an injectable dextran-based biomaterial. Spatial control is demonstrated by conformally tethering a contra-directional gradient of desthiobiotinylated and biotinylated moieties to a 3D hydrogel construct. Temporal control and cytocompatibility are demonstrated by reversibly sequestering growth factors from reporter cells using desthiobiotinylated V_H_H type antibodies, which could be on-demand displaced by pristine biotin. Post-functionalizing hydrogel constructs with biotinylated fluorophores, peptides, nanoparticles, enzymes, and antibodies confirms the universality of the competitive supramolecular complexation strategy.

## Results

### Supramolecular displacement of desthiobiotin by biotin

**Fig. 1:**
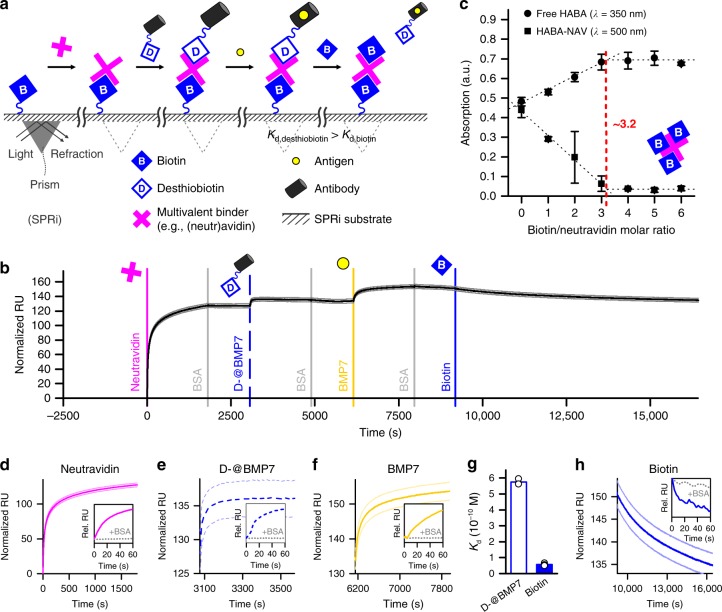
Reversible functionalization via competitive supramolecular complexation. **a**, **b** Surface plasmon resonance imaging (SPRi) was used to monitor the (single) reversible functionalization of a biotinylated 2D substrate through supramolecular complexation of multivalent (i.e., with multiple binding pockets) (neutr)avidin and desthiobiotinylated antibody. **c** The multivalent nature of neutravidin was confirmed using spectrophotometric absorption analysis of 4′-hydroxyazobenzene-2-carboxylic acid bound to neutravidin (HABA-NAV; *λ* = 500 nm), which was displaced by biotin resulting in unbound (i.e., free) HABA (*λ* = 350 nm). **b**, **d**–**f** Following a cascade binding strategy, neutravidin, desthiobiotinylated antibody against bone morphogenetic protein 7 (D-@BMP7), and BMP7 were sequentially complexed onto the biotinylated SPRi substrate. **g** Based on a difference in binding affinity, **h** supramolecular displacement of D-@BMP7 by biotin was achieved, resulting in the release of the antibody/antigen complex. The insets show the averaged response curves of a specific analyte injection versus a non-specific analyte (i.e., BSA) injection. All magenta data indicate (neutr)avidin. All blue/white dashed data indicate D-@BMP7. All yellow data indicate BMP7. All blue solid data indicate biotin. All light-colored data indicate ± standard deviation (*n* = 6). All error bars indicate ± standard deviation (*n* = 6)

The competitive binding of desthiobiotin and biotin to multivalent neutravidin was assessed using surface plasmon resonance imaging (SPRi) (Fig. 1a). Neutravidin is a deglycosylated avidin analog with similar biotin-binding affinity, but a closer to neutral isoelectric point (pI_avidin_ = 10 versus pI_neutravidin_ *=* 6), which minimizes non-specific interactions at neutral pH values^29^. Flowing a neutravidin containing solution over a pre-biotinylated SPRi substrate confirmed neutravidin complexation with biotin on a 2D material, as indicated by a significant signal increase (Fig. 1b, d and Supplementary Fig. 1). This complex was characterized by a dissociation constant *K*_d_ = 5.7 × 10^−11^ ± 0.9 × 10^−11^ M (i.e., average ± standard deviation; *n* = 3) (Supplementary Table 1), which is weaker than the biotin/avidin complex in solution (*K*_d_ ~10^−15^ M), but similar to previously reported (neutr)avidin binding affinity with substrate-tethered biotin (*K*_d_ ~10^−10^ to ~10^−12^ M)^30–32^. Flowing a bovine serum albumin (BSA) containing solution over the substrate did not increase the SPRi signal intensity, confirming that the biotin/neutravidin complexation was specific (Fig. 1d, inset).

Neutravidin’s tetrameric structure has up to four binding pockets available for the supramolecular complexation of biotinylated and desthiobiotinylated molecules^26^. The multivalent binding capacity of neutravidin was confirmed using a HABA/biotin displacement assay, which proved that neutravidin and biotin could form supramolecular complexes up to a 3.2:1 molar ratio (Fig. 1c).

Neutravidin’s multivalent nature allowed for the engineering of a biotin/neutravidin/(desthio)biotin-binding cascade, which offers a universal strategy to functionalize biotinylated biomaterials with (desthio)biotinylated molecules of interest. To demonstrate this concept, we selected desthiobiotinylated antibodies for their intrinsic capability to sequester or present specific bioactive moieties such as growth factors to guide cell behavior. Specifically, a variable domain of single chain heavy chain only antibody (V_H_H) that binds bone morphogenetic protein (BMP) 7^33^, which was functionalized with desthiobiotin (D-@BMP7) was used as a model antibody. SPRi analysis confirmed that flowing a D-@BMP7 containing solution over a biotin/neutravidin complex presenting surface resulted in the formation of a larger supramolecular complex as visualized by a strong signal increase (Fig. 1b, e). Further signal increase was observed upon injection of a BMP7 containing solution, which implied that the tethered D-@BMP7 was able to capture its target antigen (Fig. 1b, f). Both the tethering and ligand binding of D-@BMP7 were specific interactions, as confirmed by the absence of a SPRi signal increase following BSA injections (Fig. 1e, f, insets).

Binding kinetics analysis revealed that the binding between D-@BMP7 and neutravidin was an order of magnitude weaker than the binding between biotin and neutravidin (Fig. 1g and Supplementary Table 1). This difference in binding affinities was leveraged to achieve on-demand release of desthiobiotinylated moieties via supramolecular displacement of desthiobiotin by biotin. Flowing a free (i.e., unbound) biotin containing solution over the biotin/neutravidin/D-@BMP7 complex presenting substrate triggered the release of the D-@BMP7/BMP7 complex, as evidenced by a reduction in SPRi signal intensity (Fig. 1b, h). Specificity of the desthiobiotin/biotin displacement was confirmed by flowing BSA over the SPRi substrate (Fig. 1h, inset). Together, these experiments demonstrated the feasibility of temporal modifications on 2D nanocoated material surfaces via formation and competitive displacement of supramolecular complexes.

### Biotinylated hydrogels enable supramolecular modifications

**Fig. 2:**
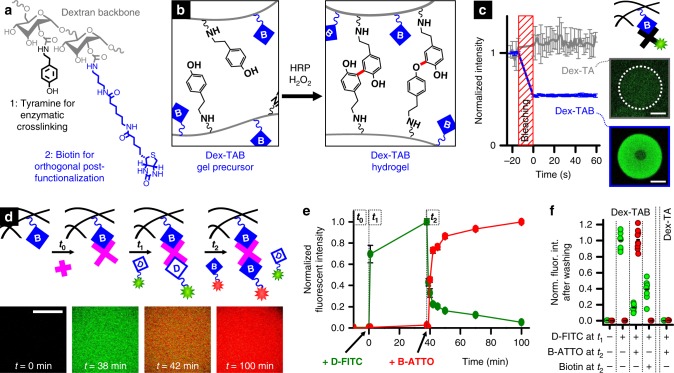
Temporal control over biotinylated hydrogel. **a** Dextran was modified with tyramine and biotin to yield the injectable polymer Dex-TAB. **b** The phenolic hydroxyl groups in Dex-TAB could be enzymatically crosslinked in situ via the formation of C–C and C–O bonds (red bonds) using horseradish peroxidase (HRP) as catalyst and H_2_O_2_ as oxidizer. This effectively resulted in a dextran-based hydrogel with biotin available for orthogonal post-functionalization. **c** Dex-TAB (blue) and not Dex-TA (gray) could be post-functionalized with fluorescent streptavidin-FITC in a stable manner, as demonstrated using fluorescence recovery after photobleaching (FRAP). **d** On-demand supramolecular complexation and in situ displacement of biochemical moieties within Dex-TAB was demonstrated using D-FITC, B-ATTO, and pristine biotin (**e**, **f**), which was quantified using time-lapse fluorescence confocal microscopy analysis. All green data indicate D-FITC. All red data indicate B-ATTO. All error bars indicate ± standard deviation (*n* = 4). All scale bars indicate 10 µm

A biotinylated injectable polymer was then synthesized to allow the fabrication of complex 3D materials with a temporally tunable biochemical composition. Dextran was selected as a bio-inert, biocompatible, and easily modifiable polymer^34,35^, thereby acting as a model template material for biotin conjugation. Besides biotin, tyramine was selected as a reactive side group to enable in situ enzymatic crosslinking^36^, which yielded a dual orthogonal injectable hydrogel readily compatible with additive manufacturing and tissue engineering strategies (Fig. 2a). The biotinylated injectable hydrogel was synthesized by functionalizing dextran with tyramine and 1,4-butanediamine groups (i.e., Dex-TA-NH_2_) (Supplementary Fig. 2) and functionalized with an amine-reactive biotin that contained a long-chain spacer (biotin-LC-NHS). Successful Dex-TA-biotin (Dex-TAB) synthesis was confirmed using ^1^H NMR (Supplementary Fig. 3). The numbers of conjugated tyramine and biotin moieties per 100 dextran anhydroglucose rings were 13 and 6, respectively, as determined by the ratios of the integrated signals of dextran (δ 4.0–5.8 ppm) and tyramine (δ 6.66 and δ 6.98 ppm), and those of tyramine and the coupled 6-aminocaproic spacer (δ 2.13 ppm). Dex-TAB could be crosslinked in situ via the formation of covalent tyramine-tyramine bonds using horseradish peroxidase (HRP) as a catalyst and H_2_O_2_ as an oxidizer (Fig. 2b and Supplementary Fig. 4).

After forming the Dex-TAB hydrogel by enzymatic crosslinking, the biotin moieties remained available for orthogonal post-functionalization via biotin/avidin complexation. This concept was demonstrated by functionalizing the Dex-TAB hydrogel with the fluorescein-labeled avidin analog streptavidin (streptavidin-FITC). Fluorescence confocal microscopy and fluorescence recovery after photobleaching (FRAP) revealed that streptavidin-FITC could be coupled to Dex-TAB hydrogels, but not to pristine dextran-tyramine (Dex-TA) hydrogels, which confirmed the availability and functionality of biotin in enzymatically crosslinked Dex-TAB hydrogels (Fig. 2c). Furthermore, Dex-TAB could be functionalized with biotinylated molecules of interest by using neutravidin as a multivalent binder. Specifically, Dex-TAB, but not Dex-TA hydrogel, allowed for functionalization via supramolecular complexation as demonstrated using sequential incubation with neutravidin and fluorescein-labeled biotin (B-FITC) (Supplementary Fig. 5). The level of functionalization linearly correlated (*R*^2^ = 0.99) with the concentration of biotin in the hydrogel (Supplementary Fig. 6). This dual orthogonal post-functionalization strategy enabled the tuning of the hydrogel’s biochemical properties without significantly (*p* > 0.1; one-way ANOVA with Tukey’s post hoc test on normally distributed data as indicated by Shapiro-Wilk *p* > 0.1) altering the hydrogel network properties, as confirmed by permeability analysis using fluorescently labeled dextran molecules (Supplementary Fig. 7).

### Temporal control over biotinylated hydrogels

It was confirmed that the Dex-TAB hydrogel could be temporally modified via the competitive binding of desthiobiotin and biotin to multivalent neutravidin. Specifically, Dex-TAB hydrogel constructs were endowed with an excess of neutravidin, which complexed with the biotin that was covalently bound to the hydrogel. The hydrogels were washed (*t*_0_) and continually imaged using fluorescence confocal microscopy to visualize and quantify the desthiobiotin binding and displacement in time (Fig. 2d, e). Incubation with desthiobiotin-FITC (D-FITC) (*t*_1_) stained the hydrogel homogeneously green within a few minutes, indicating rapid diffusion and effective binding of D-FITC to the remaining free binding pockets of the tethered neutravidin. After 40 min of incubation, the unbound fluorophores were removed by thoroughly washing the hydrogels with phosphate-buffered saline (PBS), after which red biotin-atto565 (B-ATTO) solution was added (*t*_2_). After one hour of competitive complexation, the D-FITC intensity had dropped to ~5% of its original intensity, indicating the presence of B-ATTO within the crosslinked 3D hydrogel network. Unbound fluorophores were again removed from the hydrogels by thoroughly washing the constructs. Subsequent fluorescence confocal imaging revealed that approximately 80% of the D-FITC had been replaced by B-ATTO (Fig. 2f). Importantly, no green and red fluorescent signals could be detected in Dex-TA hydrogels treated with both D-FITC and B-ATTO, which corroborated the neutravidin-mediated binding of D-FITC and B-ATTO to Dex-TAB. Furthermore, to ensure that the reduction of green fluorescent signal upon addition of B-ATTO was not merely the result of Förster resonance energy transfer (FRET) from the FITC to the ATTO fluorophore^37^, D-FITC functionalized hydrogels were also treated with non-fluorescent biotin. Pristine biotin replaced over 60% of the D-FITC within an hour, which validated the functionality of the competitive supramolecular complexation method. Longer incubation with biotin revealed over 70% displacement of D-FITC after 21 h, which did not significantly (*p* > 0.1; one-way ANOVA with Tukey’s post-hoc test on normally distributed data as indicated by Shapiro-Wilk *p* > 0.1) change after 44 and 207 h (~8 days) of incubation (Supplementary Fig. 8).

### Spatial control over biotinylated hydrogels

**Fig. 3:**
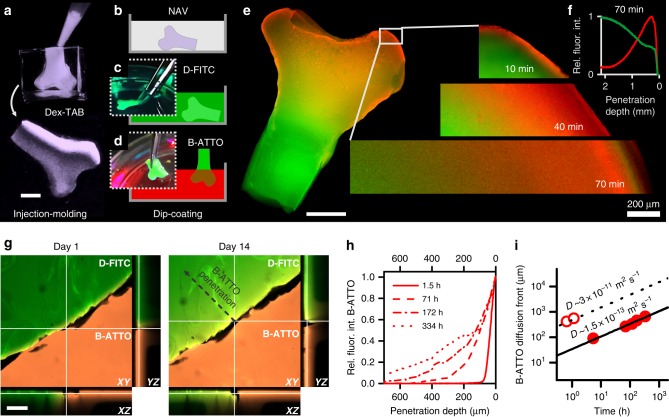
Spatiotemporal control over injectable 3D hydrogel. **a**–**f** Diffusion-controlled displacement of D-FITC bound to neutravidin (NAV) by B-ATTO using a dip-coating method revealed the spatially controlled modification of a bone-shaped injection-molded biomaterial. The smooth, conformal, and contra-directional biochemical gradients could span up to several millimeters by controlling the diffusion of competitively binding biotinylated moieties. Scale bars indicate 2 mm, unless otherwise indicated. **g** The stability of supramolecular biotin/NAV/desthiobiotin and biotin/NAV/biotin complexation within Dex-TAB was assessed by confocal time-lapse analysis of a fluorescently patterned hydrogel construct. Scale bar indicates 200 µm. **h** D-FITC did not displace B-ATTO, but B-ATTO could replace or occupy D-FITC-labeled Dex-TAB, as revealed by the progressively moving B-ATTO diffusion front. **i** The measured diffusion of B-ATTO in Dex-TAB during functionalization (open circles) matched with its theoretically predicted diffusion (dashed line; *D* = 3 × 10^−11^ m^2^ s^−1^). The moving front of B-ATTO after complexation to Dex-TAB (closed circles) was approximately 200 times slower than the diffusion of unbound B-ATTO as revealed by overlaying a theoretical plot (solid line) based on *D* = 1.5 × 10^−13^ m^2^ s^−1^. All green data indicate D-FITC. All red data indicate B-ATTO

Competitive supramolecular functionalization of the hydrogel was used to create smooth conformal gradients by controlling the penetration depth of biotinylated moieties. This feat can be used to endow 3D materials with morphogen gradients that orchestrate tissue development; natural tissue complexity most commonly originates from diffusion-based mechanisms, which thus represents a biomimetic and biologically relevant biomaterial functionalization strategy that is currently underexplored^38,39^. As a proof-of-concept, a 3D bone-shaped Dex-TAB hydrogel was created using injection molding (Fig. 3a) and functionalized with FITC in a homogeneous manner via subsequent incubation in neutravidin (Fig. 3b**)** and D-FITC solutions (Fig. 3c). The duration in which diffusion and thus supramolecular displacement occurred granted spatial control over the biomaterial’s biochemical composition, as demonstrated by timed B-ATTO dip-coatings (Fig. 3d). In particular, the thickness of the biotin-displaced shell was controlled by the dip-coat incubation time and generated contra-directional biochemically functional gradients that conformally fitted the complex curvatures of the bone-shaped hydrogel (Fig. 3e).

The diffusion of molecules through hydrogel can be described by the Stokes–Einstein Eq. (1), where *k*_*B*_, *T*, *η*, and *r* are the Boltzmann constant, temperature, dynamic viscosity of the solvent, and (hydrodynamic) radius of the solute, respectively. Consequently, the depth of a gradient within a construct can be controlled by both the dip-coating time and the size (i.e., hydrodynamic radius) of the moiety of interest. The measured diffusion coefficient of B-ATTO (*D*_B-ATTO_) in the hydrogel was 3 × 10^−11^ m^2^ s^−1^, which is in accordance with the theoretical diffusion coefficient of a molecule with a similar hydrodynamic radius (*R*_H_ *=* 0.7 nm) acknowledging the free volume theory for a 5% (w/v) 16 kDa dextran-based hydrogel^40^. Cross-sectional analysis of fluorescence microscopic images confirmed that diffusion-controlled supramolecular complexation of D-FITC and B-ATTO enabled the generation of smooth contra-directional biochemical gradients spanning several millimeters within the bone-shaped Dex-TAB hydrogel (Fig. 3f). It is of note that diffusion-based material functionalization is scalable, predictable, and universally applicable to any permeable material irrespective of its transparency, as long as diffusion can occur and diffusion constants are known.

1 $$D = k_BT/(6{\mathrm{\pi }}\eta r)$$

The stability of the biochemical gradient was studied using time-lapse confocal analysis of D-FITC and B-ATTO modified Dex-TAB hydrogels, which were covalently attached via a thin intermediary layer of pristine Dex-TA hydrogel (Fig. 3g, h). Over the course of two weeks, B-ATTO progressively moved into the D-FITC-functionalized Dex-TAB hydrogel resulting in a continuously shifting B-ATTO front characterized by *D* = 1.5 × 10^−13^ m^2^ s^−1^, which thus moved ~200 times slower than the diffusional front of free (i.e., non-complexed) B-ATTO in Dex-TAB (Fig. 3i and Supplementary Fig. 9). The relatively slow moving B-ATTO front is surprising, given the fact that SPRi analysis of the neutravidin/D-@BMP7 interaction revealed a *k*_on_ of 1.1 × 10^6^ ± 4.0 × 10^4^ M^−1^ s^−1^ (i.e., average ± standard deviation; *n* = 3) (Supplementary Table 1), which was in line with similar studies^31,41^, but two to three orders of magnitude slower than the theoretical diffusion-limited rate constant (*k*_diff_) of small biotinylated moieties as calculated using a modified Smoluchowski Eq. (2)^31,41^, where *N*_*A*_ is Avogadro’s constant, *R*_αβ_ the sum of the (hydrodynamic) radii of the molecules, and *D*_β_ the solute’s diffusion coefficient. Specifically, the smallest tested functional moieties (i.e., D-FITC and B-ATTO) that were coupled to Dex-TAB via supramolecular complexation with neutravidin are characterized by a theoretical *k*_diff_ = 5 × 10^8^ M^−1^ s^−1^. We hypothesized that the unbinding kinetics of supramolecular complexes within the Dex-TAB hydrogel were notably slower than those on SPRi substrates due to the higher rebinding efficiency, which is facilitated by 3D polymer networks versus 2D surfaces. The concept of rebinding and even double binding of avidins upon increase of biotinylation degree resulting in significantly slower unbinding kinetics has also been reported by others^42^. Furthermore, replacement of B-ATTO by D-FITC was not observed, corroborating that desthiobiotin displacement by biotin is energetically favorable. Overall, coupling biochemical moieties within Dex-TAB hydrogels through (neutr)avidin-mediated complexation is thus a feasible strategy to generate long-range multifunctional biochemical gradients. The gradually and unidirectionally growing of the multifunctional gradients is orchestrated by the supramolecular binding and unbinding kinetics, which offers the ability to engineer constructs with microenvironments that emulate the temporal behavior of natural tissues.

2 $$k_{{\mathrm{diff}}} = 2{\mathrm{\pi }}R_{{\mathrm{\alpha \beta }}}D_{\mathrm{\beta }}N_A10^3$$

### Competitive displacement by distinct biotinylated moieties

**Fig. 4:**
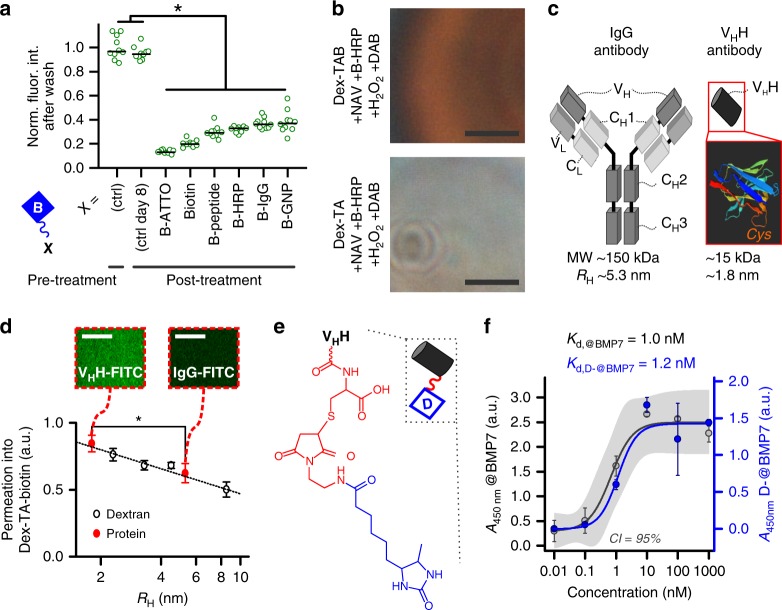
Competitive supramolecular displacement by distinct biotinylated moieties. **a** The universal nature of competitive complexation of desthiobiotinylated and biotinylated moieties with neutravidin was demonstrated by displacing D-FITC coupled to Dex-TAB by a variety of biotin-modified compounds indicated with “X” and ranked by their hydrodynamic radii (*R*_H,B-ATTO_ = 0.7 nm, *R*_H,B-GNP_ = 10 nm). “ctrl” indicates treatments with PBS. Asterisk indicates significance (*p* < 0.01; one-way ANOVA with Tukey’s post hoc test on normally distributed data as indicated by Shapiro-Wilk *p* > 0.1). **b** Supramolecularly complexed HRP enzymes remained bioactive as confirmed by the formation of brown precipitate upon addition of DAB staining solution in the presence of H_2_O_2_. B-HRP could not be supramoleculary complexed to pristine Dex-TA. **c** Single-domain (V_H_H) antibodies are an order of magnitude smaller than conventional (IgG) antibodies. **d** The relatively small hydrodynamic radius (*R*_H_) of V_H_H-type antibodies significantly improved their penetration into Dex-TAB as compared to IgG-type antibodies. Error bars indicate ± standard deviation (*n* = 4). Asterisk indicates significance (*p* < 0.05; Mann–Whitney). **e** Schematic depiction of desthiobiotinylated BMP7-binding V_H_H (D-@BMP7). **f** D-@BMP7 did not have a significantly different affinity for BMP7 as compared to the pristine BMP7-binding V_H_H (@BMP7) as confirmed by the dose-response curve of the D-@BMP7 (i.e., blue) that fits within the 95% confidence interval (CI) of @BMP7’s dose-response curve (i.e., gray). Error bars indicate ± standard deviation (*n* = 3). All scale bars indicate 10 µm

To highlight the universal nature of the competitive supramolecular complexation strategy, D-FITC-functionalized Dex-TAB was treated with a panel of widely used and readily available biotinylated moieties. To this end, we explored the complexation of biotinylated gold nanoparticles (B-GNP), peptides (B-peptide), fluorophores (B-ATTO), enzymes (B-HRP), and antibodies (B-IgG). All biotinylated moieties effectively displaced D-FITC, as witnessed by the significant (*p* < 0.01; one-way ANOVA with Tukey’s post hoc test on normally distributed data as indicated by Shapiro-Wilk *p* > 0.1) decrease of green fluorescence (Fig. 4a). D-FITC fluorescent intensity in Dex-TAB treated with PBS (i.e., “ctrl”) did not significantly change over a time span of eight days, which corroborated the relative long-term stability of desthiobiotin-mediated functionalization of biotinylated hydrogels. Moreover, the B-HRP enzymes that displaced D-FITC from the Dex-TAB/neutravidin hydrogel remained biologically functional, confirming the mild nature of the competitive supramolecular functionalization strategy (Fig. 4b). It was noted that the hydrodynamic radius of the explored biotinylated moieties was inversely correlated with the D-FITC displacement efficiency. Consequently, minimizing the biotinylated moiety’s size can thus be considered as a potent strategy to augment Dex-TAB’s post-modification efficiency and rate.

We next sought an optimal strategy to temporally control the exposure of bioactive moieties (i.e., growth factors) within 3D environments by incorporating desthiobiotinylated antibodies in hydrogels. Post-crosslinking functionalization efficiency could be controlled by selecting antibodies of various sizes. Specifically, V_H_H antibodies are smaller (molecular weight (MW) = 15 kDa; *R*_H_ = 1.8 nm) than conventional antibodies (MW = 150 kDa; *R*_H_ = 5.3 nm) (Fig. 4c), while offering similar antigen specificity and binding affinities^43^. Use of V_H_Hs was thus expected to facilitate biomaterial modifications due to their relatively small hydrodynamic radius and hence rapid diffusion properties. V_H_Hs indeed permeated the hydrogel network significantly (*p* < 0.05; Mann-Whitney) more efficient than conventional IgG-type antibodies, as confirmed using confocal analysis of fluorescently labeled V_H_H and IgG type antibodies (Fig. 4d).

A key advantage of the supramolecular displacement strategy is its capability to reversibly expose cells to stimulatory biochemical cues such as growth factors. As proof of principle, we aimed to reversibly trigger cell responses by controlling the bioavailability of growth factors in Dex-TAB. To this end, a BMP7-binding V_H_H (@BMP7)^33^ was desthiobiotinylated (D-@BMP7) by conjugating desthiobiotin-maleimide to an unpaired cysteine at the @BMP7’s C-terminus (Fig. 4e and Supplementary Fig. 10). Desthiobiotinylation of the @BMP7 had no significant effect on its affinity for BMP7, as confirmed by dose-response analysis using an enzyme-linked immunosorbent assay (ELISA) (Fig. 4f).

### Temporally controlling biomaterials to steer cell behavior

**Fig. 5:**
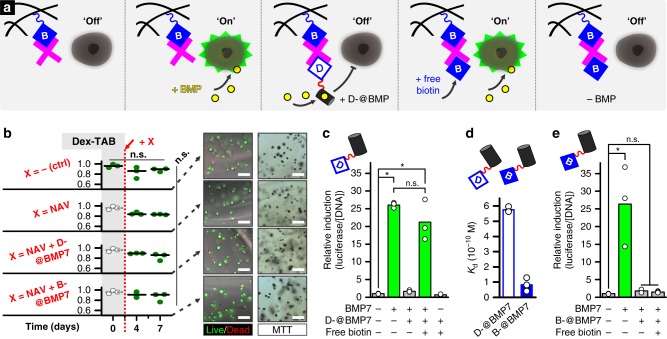
Temporally controlling cell behavior using on-demand supramolecular displacement. **a** Dex-TAB was reversibly functionalized with BMP7-binding desthiobiotinylated V_H_H (D-@BMP7) to demonstrate the temporally controlled sequestration of BMP7 as visualized using BMP reporter cells. **b** Encapsulating cells in Dex-TAB did not significantly affect cell viability (i.e., live/dead) on the short-term (0 days) and long-term (7 days). Furthermore, cell viability and metabolic activity (i.e., MTT) were not significantly affected by the post-modification of Dex-TAB with neutravidin, D-@BMP7, and B-@BMP7. “n.s.” indicates no significance (*p* > 0.1; Kruskal-Wallis ANOVA test). **c** Bioluminescence analysis of the BMP reporter cells confirmed the reversible sequestration of BMP7 by Dex-TAB hydrogel through its on-demand competitive supramolecular complexation with neutravidin, D-@BMP7, and free biotin. “n.s.” indicates no significance (*p* > 0.1). Asterisk indicates significance (*p* < 0.05; Kruskal-Wallis ANOVA test). **d** Due to its stronger affinity with neutravidin than D-@BMP7, **e** the growth factor sequestering effect of biotinylated BMP7-binding V_H_H (B-@BMP7) could not be reversed by free biotin supplementation, thereby confirming the specificity of the desthiobiotin/biotin-based supramolecular displacement mechanism. “n.s.” indicates no significance (*p* > 0.1). Asterisk indicates significance (*p* < 0.05; Kruskal-Wallis ANOVA test). All scale bars indicate 100 µm

To visualize temporal control over cell behavior via the tunable bioavailability of BMP7, Dex-TAB hydrogels were seeded with C2C12 cells that were genetically encoded to act as BMP7-responsive BRE-Luc reporters (Fig. 5a and Supplementary Fig. 11)^44^. The enzymatic crosslinking as well as the orthogonal post-functionalization of Dex-TAB with neutravidin, desthiobiotinylated, and biotinylated V_H_Hs proved cytocompatible. Specifically, these processes did not significantly (*p* > 0.1; Kruskal-Wallis ANOVA test) affect the short-term (0 days) and long-term (7 days) viability of encapsulated cells as compared to cell viability immediately after encapsulation in pristine Dex-TAB hydrogels (96% ± 3%) (i.e., average ± standard deviation; *n* = 3) (Fig. 5b). Furthermore, encapsulated cells remained metabolically active under all conditions for at least seven days. To study reversible biofunctionalization, Dex-TAB hydrogels were modified with neutravidin and sequentially exposed to different combinations of D-@BMP7, biotin, and BMP7 (Supplementary Fig. 12). Subsequently, BRE-Luc reporter cells were cultured with the prepared constructs to assess their effect on the BMP activity level (Fig. 5c). As expected, the bioluminescent signal of the reporter cells was significantly (*p* < 0.05; Kruskal-Wallis ANOVA test) increased upon BMP7 supplementation. Functionalizing the hydrogel via supramolecular complexation with neutravidin/D-@BMP7 effectively inactivated BMP7 via growth factor sequestration, as indicated by the diminishing of bioluminescence to background intensity levels. Addition of free biotin fully reinstated the BMP7-induced bioluminescence, which indicated the compatibility of supramolecular displacement of D-@BMP7 by biotin with biological processes and bioactivity of molecules. Lastly, BMP7 depletion nullified the cells’ reporter activity. This material modification strategy based on competitive supramolecular complexation thus allowed for the reversible exposure of cells to biochemical cues. Moreover, the approach uniquely enabled the on-demand reversible neutralization of specific growth factors in a chemically complex fluid by coupling antibodies to a 3D hydrogel via biotin-labile supramolecular interactions.

The specificity of the desthiobiotin/biotin displacement strategy was reconfirmed by coupling biotinylated BMP7-binding V_H_H (B-@BMP7) to Dex-TAB and exposing the modified construct to free biotin. Similar to desthiobiotinylation, biotinylation of @BMP7 had no significant effect on its affinity for BMP7, as confirmed by a dose-response analysis (Supplementary Fig. 13). SPRi revealed that B-@BMP7 bound stronger to neutravidin than D-@BMP7 (Fig. 5d and Supplementary Table 1), which prevented reporter cell activation through BMP7 sequestration when coupled to Dex-TAB and cultured with BMP7 reporter cells (Fig. 5e). Importantly, no significant (*p* > 0.1; Kruskal-Wallis ANOVA test) increase in the cellular response (i.e., bioluminescence) was measured after addition of free biotin, indicating that the complexed B-@BMP7 was not efficiently displaced by supplemented biotin. Together, these results demonstrate that supramolecular complexation of desthiobiotinylated moieties to biotinylated materials such as Dex-TAB can be effectively and specifically reversed at least once using biotin, for example, to temporally control the biochemical functionality of a material.

Besides the desthiobiotin/biotin pair, competitive complexation of functional moieties could, in principle, also be achieved by leveraging reversible interactions between avidin and alternative biotin analogs such as thiobiotin (*K*_d_~10^−12^ M), desthiobiotinol (*K*_d_ ~10^−10^ M), hexyl imidazolidone (*K*_d_~10^−9^ M), and iminobiotin (*K*_d_~10^−7^ M)^26^. This competitive supramolecular complexation strategy might therefore also support multi-stage reversible functionalization of (bio)materials by leveraging a variety of avidin and biotin analogs. In this work, we primarily focused on the complexation of neutravidin with desthiobiotin and biotin compounds as they interact strongest with avidin, which enabled the biochemical functionalization of materials that remained relatively stable for numerous days, which matches with the desired timeframe of most biological and life science applications.

## Discussion

In summary, we have successfully demonstrated the spatiotemporal functionalization of (bio)materials with biochemical moieties using the competitive complexation of desthiobiotin and biotin with multivalent avidin analogs. Specifically, we have developed and functionally characterized a biotinylated injectable polymer, called Dex-TAB. Dex-TAB could be enzymatically crosslinked and orthogonally post-functionalized with desthiobiotinylated molecules of interest in a cytocompatible manner via supramolecular complexation with (neutr)avidin. Dex-TAB was used to form complex 3D biomaterial constructs, which were spatiotemporally post-functionalized with smooth, conformal, and contra-directional biochemical gradients that spanned up to several millimeters. Spatially controlled hydrogel modification was achieved through diffusion-mediated competitive supramolecular complexation. Multi-step modification of Dex-TAB in the presence of live reporter cells revealed the possibility of the supramolecular desthiobiotin/biotin displacement strategy to provide biotinylated hydrogels with temporally controlled biochemical cues to instruct cell behavior. Given that most molecules are readily and commercially available as biotinylated and desthiobiotinylated moieties, conjugating biotin to a crosslinkable polymer is the only prerequisite to establish a biomaterial that is compatible with spatiotemporal modification via supramolecular complexation with multivalent avidin. The straightforward, cytocompatible, and readily available nature of this competitive supramolecular complexation strategy for spatiotemporal control over biomaterials is thus primed for its widespread integration in numerous (bio)material applications including additive manufacturing, injection therapies, and tissue engineering.

## Methods

### Materials

4′-Hydroxyazobenzene-2-carboxylic acid (HABA), biotin, N-(2-aminoethyl)maleimide trifluoroacetate salt (amino-maleimide), acetic acid, sodium acetate, phosphorous acid, magnesium chloride, Tween 20, Tween 80, bovine serum albumin (BSA), dimethyl sulfoxide (DMSO), dextran (Dex; MW 15–25 kg mol^−1^—*M*_n_ 16 kg/mol; lyophilized before use), 4-nitrophenyl chloroformate (PNC; sublimated before use), LiCl (dried at 110 °C before use), tyramine (TA), anhydrous pyridine, anhydrous *N*,*N*-dimethylformamide (DMF), *N*,*N*-Diisopropylethylamine (DIPEA), piperidine, amino acids, acetic anhydride, triisopropylsilane, trifluoroacetic acid (TFA), sodium hydroxide (NaOH), *N*-Boc-1,4-butanediamine (NH_2_-Boc), sodium bicarbonate (NaHCO_3_), biotin-atto565, biotin-4-fluorescein (biotin-FITC), 6-aminofluorescein, horseradish peroxidase (HRP, type VI), biotin-HRP, hydrogen peroxide (H_2_O_2_; with inhibitor), fetal bovine serum (FBS), calcein AM, ethidium homodimer-1 (EthD-1), Thiazolyl Blue Tetrazolium Blue (MTT), and all other solvents, unless otherwise stated, were purchased from Sigma-Aldrich. The bone-shaped master was purchased from LEGO. Polydimethylsiloxane (PDMS; Sylgard 184) was purchased from Dow Corning. Phosphate-buffered saline (PBS) was purchased from Lonza. Recombinant human BMP7 (354-BP-010), 3,3′,5,5′-tetramethylbenzidine (TMB), and H_2_SO_4_ were purchased from R&D Systems. Fmoc-Rink 4-methylbenzhydrylamine (MBHA) resin (50 mg scale, substitution 0.52 mmol g^−1^) and *N*,*N*,*N*′,*N*′-Tetramethyl-O-(1H-benzotriazol-1-yl)uronium hexafluorophosphate (HBTU) were purchased from MultiSynTech. Chloroform and 1-methyl-2-pyrrolidinone (NMP) were purchased from WR Chemicals. V_H_H antibody against BMP7 (Q32c-lab, clone G7) and polyclonal rabbit antibody against V_H_H (K1216) were purchased from QVQ. Biotinylated IL-1β antibody (508301, clone JK1B2, RRID:AB_315512) was purchased from Biolegend. Biotinylated IgG (Biotin-SP AffiniPure Donkey Anti-Rabbit IgG; 711-065-152) was purchased from Jackson Immunoresearch. HRP-conjugated secondary goat antibody against rabbit (P0448) was purchased from Dako. Biotinylated gold nanoparticles (20 nm) were purchased from Cytodiagnostics. The biotinylated peptide with amino acid sequence KGLPLGNSH was purchased from Pepscan. Succinimidyl 6-(biotinamido)hexanoate (biotin-LC-NHS) was purchased from ApexBio. Neutravidin, N-hydroxysuccinimide-desthiobiotin (EZ-Link NHS-desthiobiotin), 4′,6-diamidino-2-phenylindole (DAPI), and 3,3′-diaminobenzidine (DAB) staining kit were purchased from ThermoFisher Scientific. Pre-activated sensors for amine coupling (G-type easy2spot) were purchased from Ssens BV. Dulbecco’s Modified Eagle’s Medium (DMEM), Penicillin and Streptomycin, and trypsin-EDTA were purchased from Gibco. Reporter Lysis Buffer (E397A), luciferase assay reagent (E1483), and QuantiFluor dsDNA System were purchased from Promega. C2C12-BRE-Luc cells were kindly provided by prof. Daniel B. Rifkin.

### Neutravidin multivalency analysis

Spectrophotometric analysis of HABA was used to analyze the number of biotin-binding pockets of neutravidin. Specifically, HABA can specifically bind to (neutr)avidin in a similar fashion as biotin, but with lower binding affinity. The HABA/neutravidin complex has an absorption peak around 500 nm. Unbound HABA has an absorption peak around 350 nm. HABA displacement by biotin can thus be evaluated by measuring the absorbance at 350 and 500 nm. To determine neutravidin’s number of biotin-binding pockets, it was first incubated with an excess of HABA, so that fully saturated HABA/neutravidin complexes were formed. Biotin was added to these complexes in a neutravidin/biotin molar ratio of 1:1, 1:2, 1:3, 1:4, 1:5, and 1:6. For each neutravidin/biotin ratio, the amount of both the HABA/neutravidin complex and the free HABA was determined by measuring the absorbance at 500 and 350 nm, respectively, using a ND1000 spectrophotometer (ThermoFisher scientific). The plateau at 3.2 biotin/neutravidin indicated the number of biotin-binding pockets, which was in accordance with the manufacturer’s specification sheet (3.1 biotin/neutravidin).

### SPRi measurements

Biotinylated IL-1B was immobilized onto G-type easy2spot sensors by using a continuous flow spotter (Wasatch Microfluidics). Gel-type sensors were used for their increased binding capacity and more efficient use of the evanescent field, compared to a planar surface sensor. The immobilization reaction was performed in 50 mM acetic acid buffer (150 µl per spot) with antibody concentrations of 5, 2.5, 1.25, and 0.625 µg ml^−1^. As a control BSA was spotted at a concentration of 5 µg/ml. An immobilization buffer (pH 4.6) provided antibody coupling with the highest retained activity and was therefore used for all experiments. To reduce non-specific interactions, the sensor was deactivated with 1% (w/v) BSA in 50 mM acetate buffer (pH 4.6) for 7 min and subsequently with 0.2 M ethanolamine (pH 8.5) for an additional 7 min. SPRi was performed using a MX96 system with SUIT operation software (IBIS). Neutravidin, D-@BMP7, B-@BMP7, and BMP7 were dissolved in system buffer consisting of PBS with 0.5 % (w/v) BSA and 0.075% (v/v) Tween80 at a concentration of 250, 100, 100, and 100 nM, respectively. Biotin was dissolved at a concentration of 400 µM in system buffer supplemented with 0.1% (v/v) DMSO. Back-and-forth flow was set to 10 µl min^−^^1^ in a flow cell containing 12 µl of sample. Sprint software was used for data collection and referencing from a total of six spots per condition. Data was subsequently exported to Matlab R2015a for further analysis and quality control using custom scripts (available upon request). The dissociation constants of the supramolecular complexes were determined as follows: first, the sensor was washed using three blank (i.e., system buffer) injections to provide the background for interaction signals. Then, a cascade approach was used to determine the affinity between the components of the complex. To this end, the sensor was incubated with buffer followed by neutravidin, D-@BMP7 or B-@BMP7, BMP7, and finally biotin solution. The association time of cascade interactions was 30 min followed by 15 min dissociation. Between each injection the sensor was washed with system buffer. The association time of the displacement interaction with biotin was 180 min and was performed twice. The kinetics could be described with a simple 1:1 Langmuir interaction that can be captured with an exponential Eq. (3).

3 $${\mathrm{RU}}\left( t \right) = \frac{{{\mathrm{R}}_{{\mathrm{max}}}}}{{1 + \frac{{k_{{\mathrm{off}}}}}{{k_{{\mathrm{on}}}c}}}} \ast (1 - e^{\left( { - \left( {k_{{\mathrm{on}}}c + k_{{\mathrm{off}}}} \right) \ast t} \right)})$$

In this equation *R*_max_ is the binding capacity of the ligand (RU), *k*_*on*_ is the rate of association (M^−1^ s^−1^), *k*_off_ is the rate of dissociation (s^−1^), *c* is the analyte concentration (M), and *t* is time from start of the interaction (s). The dissociation constant *K*_d_ is defined as *k*_off_/*k*_on_. Matlab software with custom scripts was used to analyze the affinity data by fitting the Langmuir interaction. In short, the blanks were subtracted and the signal was zeroed to the first interaction. To determine the affinity of a specific interaction, first the *k*_off_ rate was determined in the dissociation phase. Subsequently, the *k*_on_ rate was determined using a fixed *k*_off_. For the displacement with biotin the *k*_off_ rate was determined in the association phase.

### Dex-TAB synthesis and characterization

Dextran was reacted with *p*-nitrophenyl chloroformate (PNC) to form p-nitrophenyl carbonate conjugates, which were then treated with primary amine-containing compounds (Supplementary Fig. 2). Dextran-p-nitrophenyl carbonate conjugates (Dex-PNC) were synthesized as follows. In a typical experiment, dextran (3.0 g, 56.3 mmol OH groups) was dissolved in DMF (200 mL, containing 2.4 g of LiCl) at 90 °C under a nitrogen atmosphere. After the dextran was dissolved, the mixture was cooled to 0 °C. Pyridine (1.5 mL, 18.6 mmol) and subsequently PNC (2.8 g, 13.9 mmol) were added to the solution in small portions while stirring and keeping the temperature at 0 °C. The reaction was allowed to proceed for one hour and the product was precipitated in cold ethanol. The precipitate was filtered and washed with ethanol and subsequently diethyl ether, and then dried in a vacuum oven. Dextran-tyramine-butylamine (Dex-TA-NH_2_) was synthesized as follows. Dex-PNC was first reacted with *N*-Boc-1,4-diaminobutane and then with tyramine. The protecting *t*-butyloxycarbonyl group was removed by reaction with TFA. In a typical experiment, Dex-PNC_28_ (1.0 g, 1.36 mmol PNC groups) was dissolved in 16 mL of DMF. *N*-Boc-1,4-diaminobutane (0.09 g, 0.48 mmol) was added under a nitrogen flow and the reaction was allowed to proceed for 15 min. Thereafter tyramine (0.20 g, 1.46 mmol) was added and the reaction was allowed to proceed for one hour. The product was precipitated in cold ethanol, the precipitate was filtered and washed with ethanol and diethyl ether, and then dried in a vacuum oven. Boc-protected Dex-TA-NH (0.85 g, 0.47 mmol) was dissolved in 10 mL of deionized water. Trifluoroacetic acid (TFA) (1 mL, 13.06 mmol) was added under a nitrogen atmosphere and stirred overnight. The reaction mixture was neutralized by a 2 M NaOH solution and purified by dialysis (1000 Da molecular weight cut-off) against a 150 mM NaCl solution for 48 h and deionized water for 24 h and then isolated by lyophilization. Dex-TA-NH_2_ was further functionalized with biotin by reacting 2.5 g l^−1^ Dex-TA-NH_2_ with a 20-fold molar excess of biotin-LC-NHS for at least 1 h in 0.1 M bicarbonate buffer (pH 8.5) (Supplementary Fig. 3). Dex-TAB was then purified and concentrated using a spin filter column with 3 kDa molecular weight cut-off. The successful syntheses of Dex-PNC, Dex-TA-NH_2_, and Dex-TAB were confirmed using ^1^H NMR (AVANCE III HD NanoBay 400 MHz, Bruker) in DMSO-d_6_ or D_2_O. The numbers of conjugated tyramine and butylamine moieties per 100 dextran anhydroglucose rings were determined by calculating the ratios of integrated signals from the dextran (δ 4.0–5.8 ppm) and the tyramine groups (δ 6.66 and δ 6.98 ppm), and those of dextran and the butylamine groups (δ 1.4–1.5 ppm), respectively. The number of conjugated biotin moieties per 100 dextran anhydroglucose rings was determined by calculating the ratio of integrated signals from the tyramine groups (δ 6.66 ppm and δ 6.98 ppm) and the coupled 6-aminocaproic spacer (δ 2.13).

### Dex-TAB crosslinking and orthogonal post-functionalization

Dex-TAB hydrogel constructs were prepared by mixing 5% (w/v) Dex-TAB, 3 U ml^−1^ horseradish peroxidase, and 0.05% (w/v) H_2_O_2_. For orthogonal post-functionalization, Dex-TAB hydrogels were consecutively incubated with 1 µM neutravidin in washing buffer that consisted of 1% (w/v) BSA in PBS, washed with washing buffer to remove unbound neutravidin, incubated with 1 µM biotinylated or desthiobiotinylated molecule of interest in washing buffer, and washed again with washing buffer to remove unbound molecules. Dex-TAB/neutravidin/desthiobiotin constructs could subsequently be exposed to biotinylated molecules of interest to create a contra-directional biochemical gradient. For fluorescence microscopy (EVOS FL), fluorescence confocal microscopy (Zeiss LSM 510 and Nikon A1+), and fluorescence recovery after photobleaching (FRAP; Zeiss LSM 510), the microgels were directly functionalized with streptavidin-FITC, or after neutravidin functionalization as described above, functionalized with biotin-atto565, biotin-FITC, and/or desthiobiotin-FITC that was produced in house by coupling desthiobiotin-NHS to 6-aminofluorescein in 1 M bicarbonate buffer (pH 8). The FRAP curves were obtained by plotting, as a function of time, the fluorescent intensity of the bleach spot minus the background normalized for the bleach-rate corrected average intensity before bleaching, where the bleach rate was determined by normalizing the sample’s fluorescent intensity besides the bleach spot normalized for its average intensity before bleaching. To characterize the desthiobiotin/biotin displacement, Dex-TAB hydrogel constructs were consecutively functionalized with neutravidin, washed with PBS, functionalized with desthiobiotin-FITC, washed with PBS, and functionalized with biotin-atto565, while imaged using fluorescence confocal microscopy. Finally, hydrogels were thoroughly washed several times with PBS to remove unbound B-ATTO and imaged using fluorescence confocal microscopy to quantify the relative amount of D-FITC that had been replaced by B-ATTO. The experiment was repeated with other biotin compounds to demonstrate the approach’s universality. Specifically, neutravidin/D-FITC functionalized Dex-TAB hydrogels were incubated and continually monitored for 8 days in the presence of 1 µM biotin, biotinylated peptide (i.e., B-peptide; amino acid sequence KGLPLGNSH), HRP (B-HRP), immunoglobulin G (B-IgG), or gold nanoparticles (B-GNP) in PBS. Samples were thoroughly washed with PBS for at least one day to remove unbound moieties before analysis of the D-FITC intensity. Biological function of the supramolecularly complexed B-HRP was assessed using a DAB staining kit following manufacturer’s protocol. 3D bone-shaped biotinylated hydrogels were created by injection molding Dex-TAB into a polydimethylsiloxane (PDMS) mold that was fabricated using a miniature bone-shaped master. Hydrogel bones were functionalized with FITC in a homogeneous manner via subsequent overnight incubation with 1 µM neutravidin, PBS, and 1 µM D-FITC solutions. After thoroughly washing with PBS, the upper parts of the bone-shaped hydrogels were incubated with 1 µM B-ATTO using a timed dip-coating and subsequently washed with PBS to remove unbound fluorophores, and analyzed using fluorescence microscopy. To study the long-term stability of supramolecular complexation, two Dex-TAB hydrogels of approximately 5 × 5 × 5 mm were exclusively labeled with D-FITC and B-FITC, thoroughly washed with PBS, and covalently bonded using an intermediary of pristine (i.e., non-labeled) Dex-TA via enzymatic crosslinking of tyramine moieties. The combined hydrogel constructs were then incubated in PBS and continually imaged using confocal fluorescence microscopy for 2 weeks to investigate the stability of desthiobiotinylated and biotinylated patterns. Cross-sectional intensities of all fluorescent images were determined using ImageJ software.

### Hydrogel network analysis

Diffusion analysis was performed to analyze the effect of post-functionalizing Dex-TAB hydrogel constructs with multivalent neutravidin on the hydrogel network properties. To this end, pristine Dex-TAB hydrogel constructs and constructs that were homogeneously post-functionalized using an excessive amount of neutravidin were combined with FITC-labeled dextran with hydrodynamic radii *R*_H_ = 2.3 nm (10 kDa), *R*_H_ = 8.5 nm (150 kDa), and *R*_H_ = 27 nm (2000 kDa). The hydrogel constructs in the fluorescent solutions were then analyzed using fluorescence confocal imaging (Zeiss LSM 510) and fluorescent intensity within and outside of the constructs was quantified using ImageJ software. The relative permeation of fluorescently labeled dextran molecules into Dex-TAB was determined by normalizing the intensity with the fluorescent intensity outside hydrogels. The same approach was used to quantify and compare the relatively permeability of 5% (w/v) Dex-TAB for FITC-labeled conventional IgG antibodies and V_H_H.

### V_H_H modification and characterization

Maleimide-conjugated desthiobiotin was synthesized by reacting amino-maleimide and desthiobiotin-NHS for 2 h in bicarbonate buffer (pH 8). Desthiobiotin-maleimide was purified using high-pressure liquid chromatography on a 2545 Quaternary Gradient Module (Waters) from 90%/10% Water/Acetonitrile (0.1% trifluoroacetic acid (TFA)) to 100% Acetonitrile (0.1% TFA). The collected product was characterized with mass spectrometry; MS(ESI): *m*/*z* = 336.96 (calculated *m*/*z* = 337.18 [M + H] for C_16_H_24_N_4_O_4_). The solvents were evaporated using a rotatory vacuum evaporator, after which the desthiobiotin-maleimide was resuspended in milliQ water, frozen in liquid nitrogen, and lyophilized overnight. Desthiobiotinylated and biotinylated V_H_H were synthesized by QVQ BV by reacting the unmodified cysteine of the V_H_H with maleimide-conjugated desthiobiotin and maleimide-conjugated biotin, respectively. We used ELISA to assess the affinity against BMP7 of non-modified versus desthiobiotinylated (D-@BMP7) and biotinylated (B-@BMP7) V_H_H. To this end, a 96-well Maxisorp plate was coated overnight at 4 °C with 0.5 µg ml^−1^ human BMP7 in PBS. The plates were blocked with 4% skimmed milk in PBS and various concentrations (0–5 µM) of V_H_H were added in 1% skimmed milk. Detection of V_H_Hs was done using a polyclonal rabbit antibody against V_H_H and an HRP-conjugated secondary goat antibody against rabbit. Addition of H_2_O_2_ together with TMB identified the amount of HRP by conversion into a colored product. H_2_SO_4_ was added to stop the reaction. Spectrophotometric absorption measurements were carried out at 450 nm using a plate reader (Multiscan GO, ThermoFisher Scientific). Dose-response curves were obtained by fitting a logistic function to the measured data using Eq. (4).

4 $$y = \frac{{{\mathrm{A}}_1 - {\mathrm{A}}_2}}{{1 + ({\mathrm{x}}/{\mathrm{x}}_0)^p}} + {\mathrm{A}}_2$$

### Cell culture

C2C12-BRE-Luc cells were cultured in culture medium consisting of 20% (v/v) FBS, 1% (v/v), 100 U/ml Penicillin, and 100 µg/ml Streptomycin in DMEM. Cells were cultured under 5% CO2 at 37 °C and medium was replaced 2 times per week. When cell culture reached near confluence, the cells were detached using 0.25% (w/v) Trypsin-EDTA at 37 °C and subsequently sub-cultured or used for experimentation. Viability of cells encapsulated in enzymatically crosslinked 5% (w/v) Dex-TAB was analyzed immediately post encapsulation (i.e., day 0) and after four and seven days of in vitro culture by incubating cells for 30 min with 2 µM calcein AM (live) and 4 µM EthD-1 (dead) in PBS, and visualizing labeled cells using fluorescence microscopy. Live/dead analysis was also performed following post-modification of cell-laden Dex-TAB hydrogels with neutravidin, D-@BMP7, and B-@BMP7 using the same post-production functionalization protocol as described in the previous methods section “Dex-TAB crosslinking and orthogonal post-functionalization”. Metabolic activity of cells was analyzed by staining cells using 0.5 g l^−1^ MTT in culture medium and subsequent visualization using brightfield microscopy. For BMP7 induction experiments, cells were seeded at 10,000 cells cm^−2^ on tissue culture plastic and cultured overnight. Cells were starved using culture medium containing 0.5% (v/v) FBS for a period of 12 h. Following starvation, prefabricated Dex-TAB/neutravidin, Dex-TAB/neutravidin/D-@BMP7, Dex-TAB/neutravidin/B-@BMP7, or biotin-treated Dex-TAB/neutravidin/D-@BMP7 hydrogel constructs were added to the cell culture, which were subsequently exposed to 100 ng ml^−1^ of BMP7 for 10 to 15 h (Supplementary Fig. 12). Subsequently, the cells were lysed using reporter lysis buffer and a single freeze–thaw cycle. Luciferase expression was determined using a luciferase assay following manufacturer’s protocol and a luminometer (Victor X3, Perkin Elmer). Luciferase expression was normalized to the total DNA content, which was quantified using the QuantiFluor dsDNA System following manufacturer’s protocol and a fluorometer (Victor X3).

### Data representation and statistics

Spectrophotometric absorption measurements of HABA were performed on six samples per condition and reported as the average ± standard deviation. SPRi measurements were performed on six spots per condition and reported as the average ± standard deviation (light-colored lines). The SPRi readouts of individual spots were normalized against the *R*_max_ of the neutravidin binding curves to correct for differences in spotting density (Supplementary Fig. 1). The *k*_on_, k_off_, and *K*_d_ of supramolecular interactions were determined from at least three SPRi experiments and reported as the average ± standard deviation or overlaying individual datapoints. FRAP measurements of fluorescently labeled streptavidin were performed on at least four hydrogel constructs per condition and reported as the average ± standard deviation normalized to the average intensity before bleaching. The fluorescence (confocal) intensity measurements (including time-lapse experiments) were performed on at least four hydrogel constructs per condition and reported as the average ± standard deviation or overlaying individual datapoints, all normalized to the highest average intensity. Linear regression analysis was performed using OriginPro 2016 software. Displacement of D-FITC by biotinylated moieties was measured using fluorescence microscopy on ten samples per condition. Significance of differences was studied using a one-way ANOVA with Tukey’s post-hoc test (data was normally distributed as indicated by Shapiro-Wilk test: *p* > 0.1). The permeation of fluorescently labeled dextran molecules, V_H_H, and IgG into Dex-TAB was measured on four hydrogel constructs per condition and reported as the average ± standard deviation. Significance of differences between V_H_H and IgG permeability was determined using a Mann–Whitney test. Live/dead quantification was performed on three cell-laden hydrogels per condition and reported as the average overlaying the individual datapoints. Significance of differences was determined using a Kruskal-Wallis ANOVA test. Dose response analyses were performed using three samples per concentration and reported as the average ± standard deviation and an overlaying fitted logistic function based on Eq. (4). Confidence intervals of the dose response curves were automatically generated by the graph plotting software. The relative induction of C2C12-BRE-Luc cells was measured on three in vitro samples per condition and reported as the average ± standard deviation. Significance of differences was determined using Kruskal-Wallis ANOVA tests.

### Schematics

All graphs were made using OriginPro 2016 software. All schematics were made using ChemDraw Professional 16.0 software and CorelDRAW X7 software.

### Reporting summary

Further information on research design is available in the Nature Research Reporting Summary linked to this article.

## Supplementary information

Supplementary information

Reporting summary

## Data Availability

The data that support the findings of this study are available from the corresponding author upon reasonable request.
