# Supplementary material for: Spatiotemporal material functionalization via competitive supramolecular complexation of avidin and biotin analogs
Source: Nat Commun. 2019 Sep 25;10:4347. doi: 10.1038/s41467-019-12390-4 (PMC6761202; doi:10.1038/s41467-019-12390-4)
Supplement: Supplementary file 1 — Supplementary information [file 41467_2019_12390_MOESM1_ESM.pdf]

Supplementary Information

Spatiotemporal Material Functionalization via Competitive Supramolecular Complexation of Avidin and Biotin Analogs

Kamperman et al.

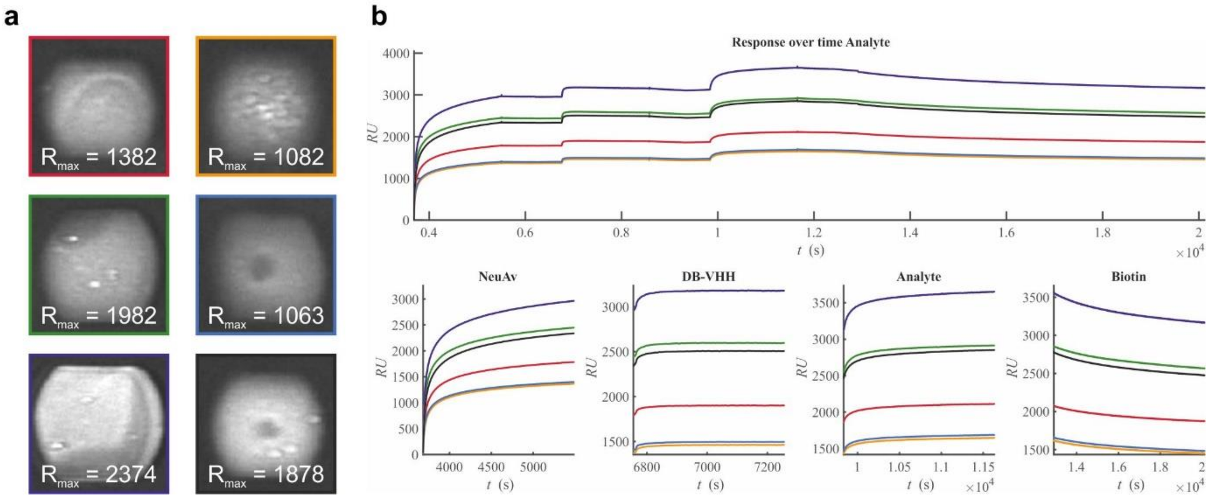

Supplementary Fig. 1. SPRi analysis of the supramolecular biotin/neutravidin/D-@BMP7 binding cascade. (**a**) Analyzed SPRi spots and R_{max} to which all SPRi data was normalized to correct for differences in spotting density. (**b**) Raw SPRi data before normalizing to spotting density.

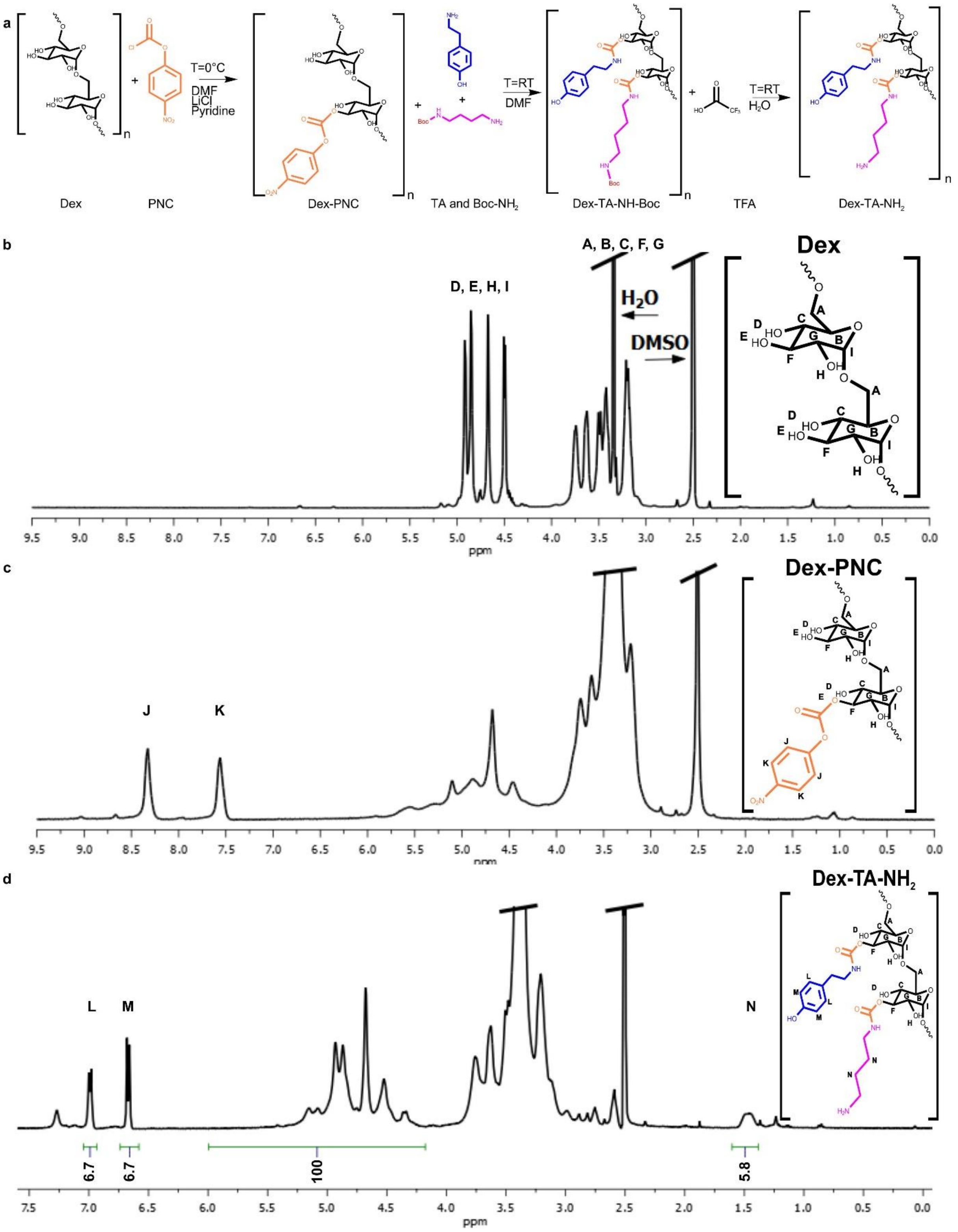

Supplementary Fig. 2. Synthesis and characterization of Dex-TA-NH_2. (**a**) Dextran (Dex) was activated with 4-nitrophenyl chloroformate (PNC), which was substituted with tyramine (TA) and Boc-protected 1,4-butanediamine (Boc-NH_2) that was subsequently deprotected using

trifluoroacetic acid (TFA). (**b-d**) ^{1}H-NMR analysis was used to confirm the successful synthesis of Dex-PNC and Dex-TA-NH_2 and to quantify the numbers of conjugated tyramine and butylamine moieties per 100 dextran anhydroglucose rings by calculating the ratios of integrated signals from anomeric and hydroxylic protons of the dextran (δ 4.0 – 5.8 ppm), and aromatic protons of the tyramine groups (δ 6.66 ppm and δ 6.98 ppm) and central methylene protons of the butylamine groups (δ 1.4 – 1.5 ppm), respectively.

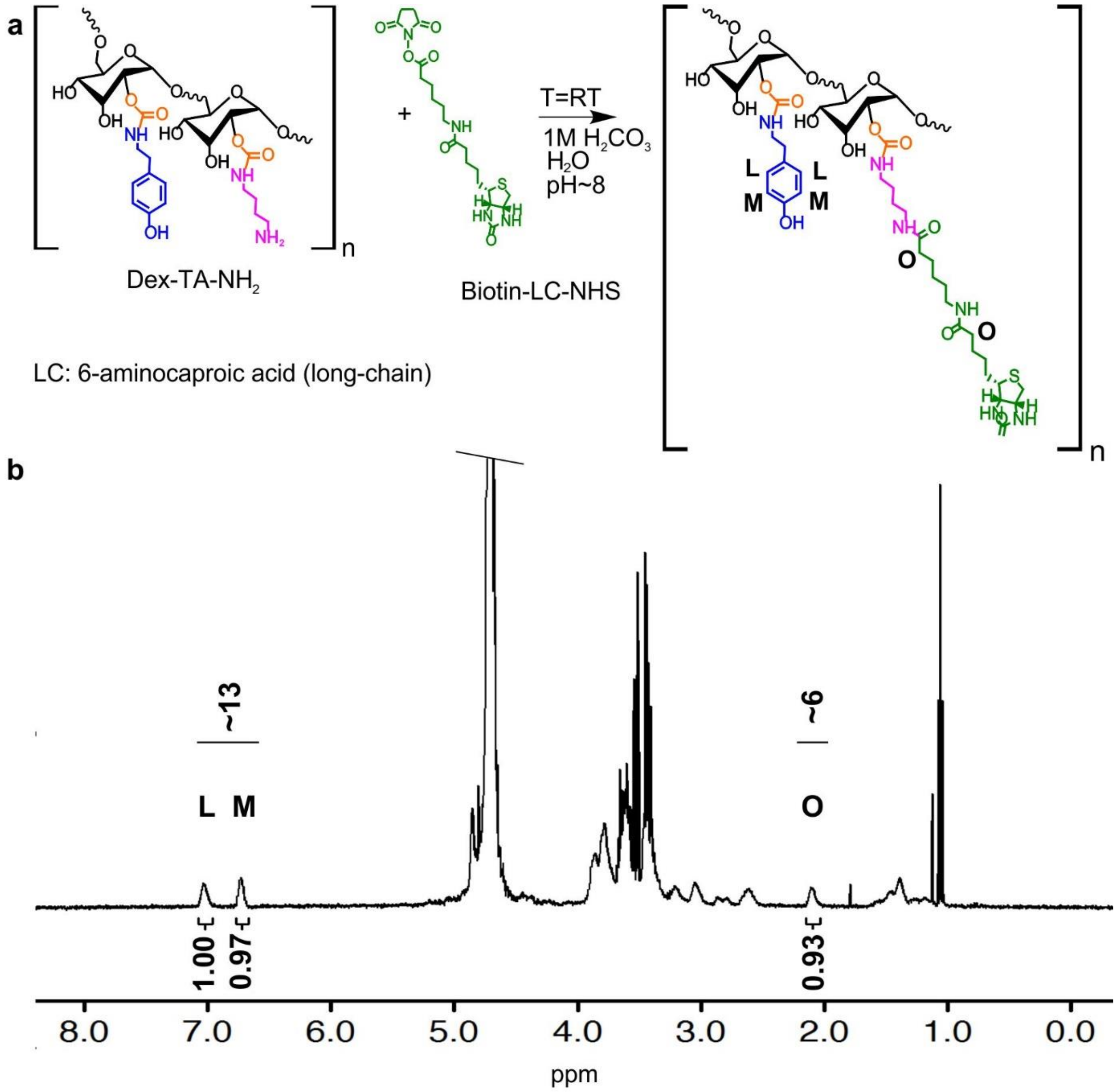

Supplementary Fig. 3. Synthesis and characterization of Dex-TAB. (**a**) Dex-TA-NH_2 was functionalized with biotin using succinimidyl 6-(biotinamido)hexanoate (biotin-LC-NHS; where LC is 6-aminocaproic acid (long-chain)). (**b**) ^{1}H-NMR analysis was used to confirm the successful synthesis of Dex-TAB and to quantify the number of conjugated biotin moieties per 100 dextran anhydroglucose rings (as determined in Supplementary Fig. 1) by calculating the ratio of integrated signals from aromatic protons of the tyramine groups (δ 6.66 ppm and δ 6.98 ppm) and carboxylic amide protons of the coupled 6-aminocaproic spacer (δ 2.13 ppm).

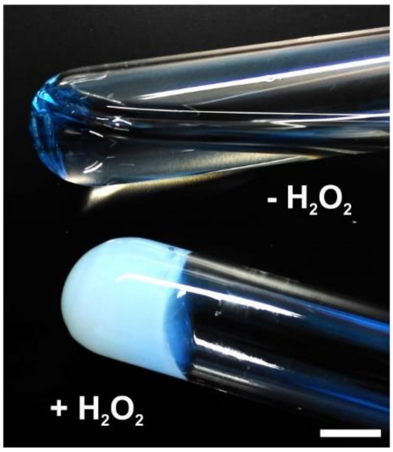

Supplementary Fig. 4. Dex-TAB gelation. Enzymatic crosslinking of Dex-TAB using HRP and H_2O_2 resulted in hydrogel network formation, as confirmed by the vial tilting method. Scale bar indicates 5 mm.

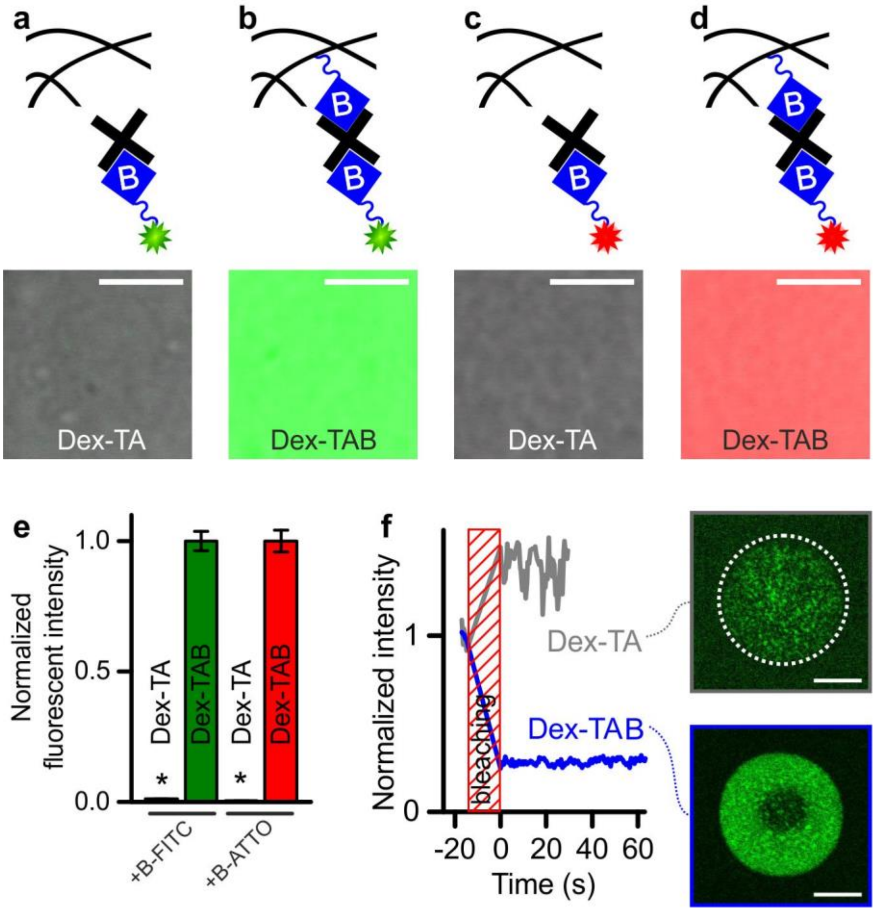

Supplementary Fig. 5. Orthogonal post-functionalization of Dex-TAB with biotinylated fluorophores via neutravidin-mediated supramolecular complexation. (**a-e**) Biotinylated dextran-tyramine (Dex-TAB) hydrogel could be post-functionalized with biotinylated FITC (B-FITC) and biotinylated atto565 (B-ATTO) using multivalent (neutr)avidin. In contrast, pristine Dex-TA could not be post-functionalized using this orthogonal biochemical post-functionalization approach, as revealed by the undetectable (indicated with '*') fluorescent signals of washed samples. (**f**) FRAP measurements of Dex-TAB (blue) and Dex-TA (grey) in the presence of neutravidin and B-FITC confirmed that biotinylated moieties stably bound to Dex-TAB and freely diffused through Dex-TA`. All green data indicate D-FITC. All red data indicate B-ATTO. All error bars indicate ± standard deviation (n = 4). All scale bars indicate 10 µm.

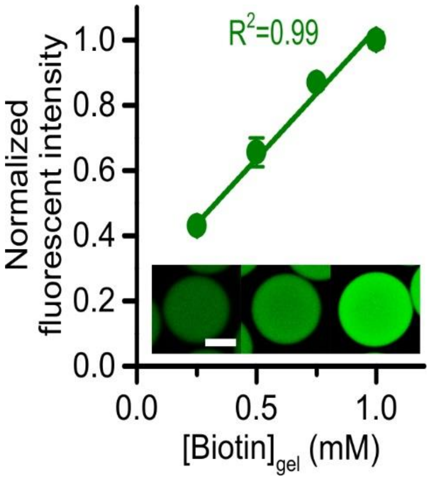

Supplementary Fig. 6. Tuning the biochemical composition of Dex-TAB. The amount of post-functionalization with biotinylated fluorophores via multivalent (neutr)avidin associated in a linear (R^2 = 0.99) manner with the concentration of biotin in the Dex-TAB hydrogel. Various biotin concentrations were achieved by mixing Dex-TAB and Dex-TA before crosslinking. All error bars indicate ± standard deviation (n = 8). Scale bar indicates 10 µm.

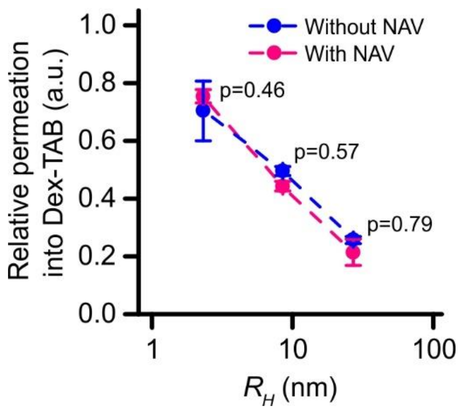

Supplementary Fig. 7. Dex-TAB hydrogel network permeability is not affected by neutravidin coupling. Permeation of fluorescently labeled dextran molecules into Dex-TAB hydrogel correlated with the dextran's hydrodynamic radius ($2.3 < R_H < 27$ nm), but did not significantly alter in the presence of neutravidin (p>0.1; one-way ANOVA with Tukey's post-hoc test on normally distributed data as indicated by Shapiro-Wilk p>0.1). All error bars indicate ± standard deviation (n ≥ 6).

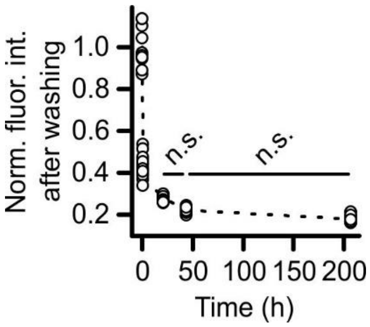

Supplementary Fig. 8. Fluorescence time lapse analysis of biotin-driven displacement of D-FITC from neutravidin-functionalized Dex-TAB. ‘n.s.’ indicates no significance ($p>0.1$; one-way ANOVA with Tukey’s post-hoc test on normally distributed data as indicated by Shapiro-Wilk $p>0.1$).

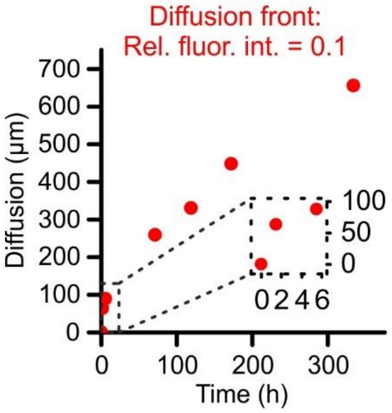

Supplementary Fig. 9. Stability of supramolecularly complexed Dex-TAB/neutravidin/D-FITC and Dex-TAB/neutravidin/B-ATTO complexes. Time-lapse analysis revealed a progressively moving B-ATTO diffusion front, which was defined as the penetration depth in the confocal cross sectional intensity plots (Fig. 3h) at which the relative fluorescent intensity was 0.1.

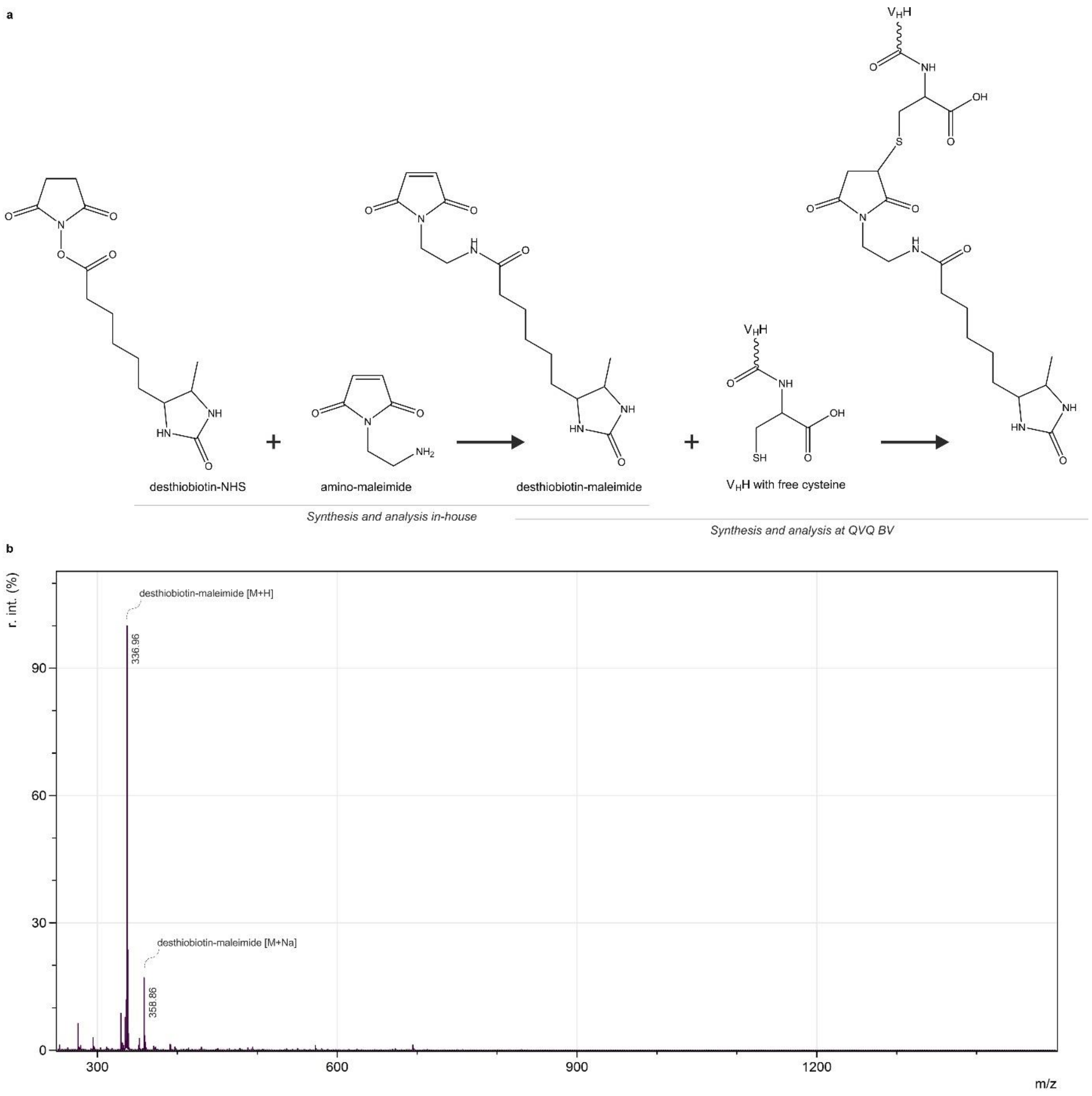

Supplementary Fig. 10. Desthiobiotinylated V_HH synthesis. (**a**) Desthiobiotin-maleimide was synthesized by coupling desthiobiotin-NHS to amino-maleimide post purification (**b**) as confirmed by liquid chromatography/mass spectroscopy (LC-MS).

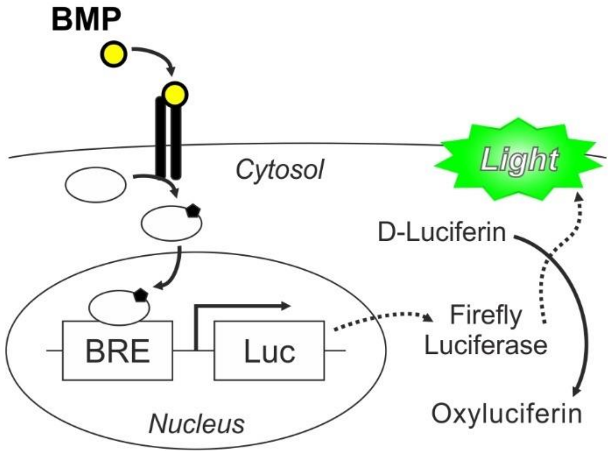

Supplementary Fig. 11. Genetically encoded reporter cells enable specific *in situ* quantification of BMP activity. The reporter cell line C2C12-BRE-Luc produces the enzyme firefly luciferase in response to BMPs, including BMP7, in a dose dependent manner. Firefly luciferase was quantified by catalyzing the transformation of D-luciferin into oxyluciferin, which resulted in bioluminescence.

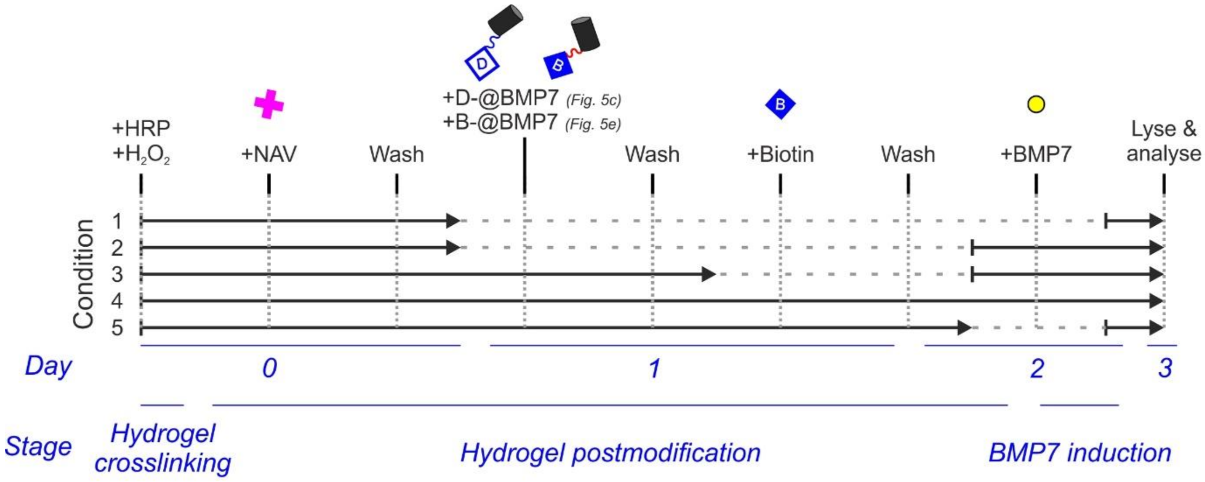

Supplementary Fig. 12. Timeline of BMP induction experiment. After enzymatic crosslinking, Dex-TAB was exposed to neutravidin (NAV), D-@BMP7, B-@BMP7, and/or biotin, after which the cell/hydrogel cultures were supplemented with BMP7 according to this scheme. Finally, all cell/hydrogel cultures (indicated by 1 to 5) were lysed and analyzed.

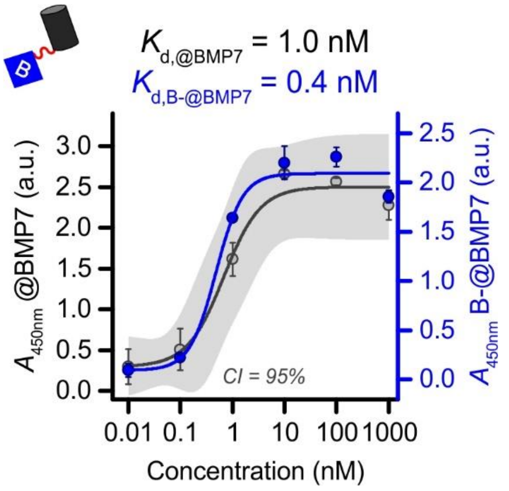

Supplementary Fig. 13. Binding of anti-BMP7 V_HH is not changed by biotinylation. Binding affinity of the V_HH against BMP7 (@BMP7) is not significantly altered by end-modifying the evolutionary conserved free cysteine with maleimide-biotin, as confirmed by the dose-response curve of the biotinylated @BMP7 (B-@BMP7; i.e., blue) that fits within the 95% confidence interval (CI) of pristine @BMP7's dose-response curve (i.e., gray). Error bars indicate ± standard deviation (n = 3).

Supplementary Table 1. Kinetic data of the supramolecular complexes. Data (i.e., average ± standard deviation; n = 3) was obtained from SPRi results by averaging three experiments with eight spots per experiment.

**Supramolecular complex**	k_{on} **(M^{-1} s^{-1})**	k_{off} **(s^{-1})**	K_d **(M)**
Soluble neutravidin with substrate-tethered biotin	$4.43\times10^{4} \pm 1.26\times10^{4}$	$2.45\times10^{-6} \pm 3.79\times10^{-7}$	$5.70\times10^{-11} \pm 8.79\times10^{-12}$
Substrate-tethered neutravidin with soluble D-@BMP7	$1.13\times10^{6} \pm 4.04\times10^{4}$	$6.49\times10^{-4} \pm 2.75\times10^{-5}$	$5.75\times10^{-10} \pm 1.81\times10^{-11}$
Substrate-tethered neutravidin with soluble B-@BMP7	$1.14 \times10^{6} \pm 1.59\times10^{5}$	$8.94\times10^{-5} \pm 3.61\times10^{-5}$	$8.28\times10^{-11} \pm 4.38\times10^{-11}$
