# Supplementary material for: Spatiotemporal material functionalization via competitive supramolecular complexation of avidin and biotin analogs
Source: Nat Commun. 2019 Sep 25;10:4347. doi: 10.1038/s41467-019-12390-4 (PMC6761202; doi:10.1038/s41467-019-12390-4)
Supplement: Supplementary file 2 — Reporting summary [file 41467_2019_12390_MOESM2_ESM.pdf]

nature research

Corresponding author(s): Dr. T. Kamperman and Dr. J. Leijten

Last updated by author(s): Aug 28, 2019

Reporting Summary

Nature Research wishes to improve the reproducibility of the work that we publish. This form provides structure for consistency and transparency in reporting. For further information on Nature Research policies, see Authors & Referees and the Editorial Policy Checklist.

Statistics

For all statistical analyses, confirm that the following items are present in the figure legend, table legend, main text, or Methods section.

n/a	Confirmed	
☐	☒	The exact sample size (*n*) for each experimental group/condition, given as a discrete number and unit of measurement
☐	☒	A statement on whether measurements were taken from distinct samples or whether the same sample was measured repeatedly
☐	☒	The statistical test(s) used AND whether they are one- or two-sided<br>*Only common tests should be described solely by name; describe more complex techniques in the Methods section.*
☒	☐	A description of all covariates tested
☐	☒	A description of any assumptions or corrections, such as tests of normality and adjustment for multiple comparisons
☐	☒	A full description of the statistical parameters including central tendency (e.g. means) or other basic estimates (e.g. regression coefficient) AND variation (e.g. standard deviation) or associated estimates of uncertainty (e.g. confidence intervals)
☐	☒	For null hypothesis testing, the test statistic (e.g. F, t, r) with confidence intervals, effect sizes, degrees of freedom and P value noted<br>*Give P values as exact values whenever suitable.*
☒	☐	For Bayesian analysis, information on the choice of priors and Markov chain Monte Carlo settings
☒	☐	For hierarchical and complex designs, identification of the appropriate level for tests and full reporting of outcomes
☐	☒	Estimates of effect sizes (e.g. Cohen's *d*, Pearson's *r*), indicating how they were calculated

Our web collection on statistics for biologists contains articles on many of the points above.

Software and code

Policy information about availability of computer code

Data collection	*Provide a description of all commercial, open source and custom code used to collect the data in this study, specifying the version used OR state that no software was used.*
Data analysis	*Provide a description of all commercial, open source and custom code used to analyse the data in this study, specifying the version used OR state that no software was used.*

For manuscripts utilizing custom algorithms or software that are central to the research but not yet described in published literature, software must be made available to editors/reviewers. We strongly encourage code deposition in a community repository (e.g. GitHub). See the Nature Research guidelines for submitting code & software for further information.

Data

Policy information about availability of data

All manuscripts must include a data availability statement. This statement should provide the following information, where applicable:

- Accession codes, unique identifiers, or web links for publicly available datasets
- A list of figures that have associated raw data
- A description of any restrictions on data availability

The data that support the findings of this study are available from the corresponding author upon reasonable request.

Field-specific reporting

Please select the one below that is the best fit for your research. If you are not sure, read the appropriate sections before making your selection.

☒ Life sciences ☐ Behavioural & social sciences ☐ Ecological, evolutionary & environmental sciences

Life sciences study design

All studies must disclose on these points even when the disclosure is negative.

Sample size	No sample-size calculation was performed. At least biological triplicates were chosen, which is consistent with studies of similar type. Statistical analysis was adjusted to the sample size. E.g., non-parametric analysis was done to study significance of biological triplicates.
Data exclusions	No data was excluded.
Replication	Most experiments were performed independently in duplo by two experimenters (student & supervisor). Results were reproducible.
Randomization	Randomization intrinsically appeared from biological triplicates and/or replication by different experimenters.
Blinding	All results are based on blinded analysis, objective automated protocols/equipment, software-based image (fluorescent intensity) analysis, or statistical analysis. Scoring/outcome was not sensitive to subjective data interpretation.

Reporting for specific materials, systems and methods

We require information from authors about some types of materials, experimental systems and methods used in many studies. Here, indicate whether each material, system or method listed is relevant to your study. If you are not sure if a list item applies to your research, read the appropriate section before selecting a response.

Materials & experimental systems

n/a	Involved in the study	
☐	☒	Antibodies
☐	☒	Eukaryotic cell lines
☒	☐	Palaeontology
☒	☐	Animals and other organisms
☒	☐	Human research participants
☒	☐	Clinical data

Methods

n/a	Involved in the study	
☒	☐	ChIP-seq
☒	☐	Flow cytometry
☒	☐	MRI-based neuroimaging

Antibodies

Antibodies used	VHH antibody against BMP7 (Q32c-lab, clone G7) and polyclonal rabbit antibody against VHH (K1216) were purchased from QVQ. Biotinylated IL-1β antibody (508301, clone JK1B2, RRID:AB_315512) was purchased from Biolegend. Biotinylated IgG (Biotin-SP AffiniPure Donkey Anti-Rabbit IgG; 711-065-152) was purchased from Jackson Immunoresearch. HRP-conjugated secondary goat antibody against rabbit (P0448) was purchased from Dako.
Validation	QVQ - Q32c-lab, clone G7<br>Source: Recombinant monoclonal VHH (Llama glama), purified from S.cerevisiae using affinity chromatography. Immunization with recombinant BMP7. Phage-display selection on immobilized BMP7 with total elution. Specificity: Human BMP7.<br>http://www.qvquality.com/wp-content/uploads/2019/08/Target-sheet-BMP7-web-jul-2019-1.pdf<br><br>Biolegend - 508301, clone JK1B2<br>Reactivity: Human. Antibody Type: Monoclonal. Host Species: Mouse. Immunogen: Recombinant human IL-1 beta.<br>https://www.biolegend.com/fr-ch/products/biotin-anti-human-il-1beta-antibody-1578

Eukaryotic cell lines

Policy information about cell lines

Cell line source(s)	C2C12-BRE-Luc cells were kindly provided by prof. Daniel B. Rifkin.
Authentication	The cell line was authenticated using a positive control experiment in which Luciferase production by the cell line was induced by recombinant human BMP7 in a dose dependent manner.
Mycoplasma contamination	The C2C12-BRE-Luc cell line has been tested for mycoplasma contamination and found to be negative (contamination-free).
Commonly misidentified lines (See ICLAC register)	N.A.
